# Hexahydrocurcumin mitigates angiotensin II-induced proliferation, migration, and inflammation in vascular smooth muscle cells

**DOI:** 10.17179/excli2023-6124

**Published:** 2023-06-05

**Authors:** Luckika Panthiya, Jiraporn Tocharus, Waraluck Chaichompoo, Apichart Suksamrarn, Chainarong Tocharus

**Affiliations:** 1 Department of Anatomy, Faculty of Medicine, Chiang Mai University, Chiang Mai 50200, Thailand; 2 Graduate School, Chiang Mai University, Chiang Mai 50200, Thailand; 3 Department of Physiology, Faculty of Medicine, Chiang Mai University, Chiang Mai 50200, Thailand; 4 Department of Chemistry and Center of Excellence of Innovation in Chemistry, Faculty of Science, Ramkhamhaeng University, Bangkok 10240, Thailand; 5 Functional Food Research Center for Well-Being, Chiang Mai University, Chiang Mai 50200, Thailand

**Keywords:** hexahydrocurcumin, angiotensin II, vascular smooth muscle cell, proliferation, migration, inflammation

## Abstract

The proliferation and migration of vascular smooth muscle cells (VSMCs) play vital roles in the pathogenesis of atherosclerosis and hypertension. It has been proposed and verified that hexahydrocurcumin (HHC), a metabolite form of curcumin, has cardiovascular protective effects. This study examined the effect of HHC on angiotensin II (Ang II)-induced proliferation, migration, and inflammation in rat aortic VSMCs and explored the molecular mechanisms related to the processes. The results showed that HHC significantly suppressed Ang II-induced proliferation, migration, and inflammation in VSMCs. HHC inhibited Ang II-induction of the increase in cyclin D1 and decrease in p21 expression in VSMCs. Moreover, HHC attenuated the generation of reactive oxygen species (ROS), and the expression of nuclear factor kappa B (NF-κB), tumor necrosis factor-α (TNF-α), interleukin-6 (IL-6) and matrix metalloproteinases-9 (MMP9) in Ang II-induced VSMCs. The proliferation, migration, inflammation, and ROS production were also inhibited by GKT137831 (NADPH oxidase, NOX1/4 inhibitor) and the combination of HHC and GKT137831. In addition, HHC restored the Ang-II inhibited expression of peroxisome proliferator-activated receptor-γ (PPAR-γ) and peroxisome proliferator activated receptor-γ coactivator-1α (PGC-1α). These findings indicate that HHC may play a protective role in Ang II-promoted proliferation, migration, and inflammation by suppressing NADPH oxidase mediated ROS generation and elevating PPAR-γ and PGC-1α expression.

See also Figure 1(Fig. 1).

## Introduction

Vascular smooth muscle cells (VSMCs) are an essential component of vascular walls, and their highly differentiated contractile phenotype is critically responsible for maintaining vascular homeostasis. The abnormal proliferation and migration of VSMCs and their expression of various pro-inflammatory cytokines are key elements in the pathologies and development of vascular diseases including atherosclerosis and hypertension (Li et al., 2017[19]; Wu et al., 2018[40]). Angiotensin II (Ang II) is one of the active components of the renin-angiotensin system contributing to the regulation of blood pressure and is also involved in the regulation of several cellular processes such as cell growth, migration, inflammation, and fibrosis (Louis and Zahradka, 2010[20]; Touyz, 2004[36]). Ang II is a potent mediator of oxidative stress and oxidant signaling. There is increasing evidence to indicate that the activation of NADPH oxidase (NOX), a major component of reactive oxygen species (ROS), is involved in many pathways of Ang II signaling in vascular cells (Nguyen Dinh Cat et al., 2013[25]; Taniyama and Griendling, 2003[35]). NADPH oxidase generated ROS contribute to increased Ang II-mediated VSMC growth in hypertension (Touyz, 2003[37]). Ang II activates redox-sensitive transcription factors, and the nuclear factor kappa B (NF- κB), which is important in the production of proinflammatory cytokines and interleukins. VSMCs are activated to migrate and proliferate in response to the increase in growth factors and cytokines such as interleukin-1 (IL-1), interleukin-6 (IL-6), tumor necrosis factor-α (TNF-α) and matrix metalloproteinases (MMPs) (Marx et al., 2011[22]). The expression of MMPs is also regulated by NF-κB and oxidative stress potentially increases the activity and expression of MMP. Uncontrolled MMP activity results in the break down of the components of ECM, enabling VSMCs to promote a migratory phenotype (Chou et al., 2019[5]).

Peroxisome Proliferator-Activated Receptor-γ (PPAR-γ), a nuclear hormone receptor superfamily, is present in the VSMCs of the vascular walls in both humans and rodents (Duan et al., 2008[9]). It is a potential protective factor in the case of vascular injury and is increasing in importance in medicine (Hsueh, 2001[11]). Previous studies reported that the activation of PPAR-γ attenuated Ang II-stimulated cell growth, migration, and inflammatory responses (Benkirane et al., 2006[1]; Duan et al., 2008[9]; Marchesi et al., 2013[21]). PGC-1α [PPARγ (peroxisome-proliferator-activated receptor γ) co-activator-1α] is a nuclear protein that regulates a range of endothelial and smooth muscle cell processes. Interestingly, it has been reported that PGC-1α is a powerful regulator of ROS metabolism and the overexpression of PGC-1α could attenuate ROS production, thus ameliorating Ang II-induced VSMC proliferation (Pei et al., 2016[29]; Zhao et al., 2015[43]). 

In this study hexahydrocurcumin (HHC), one of the major phases I metabolites of curcumin, is investigated. Several studies reported that HHC exhibits similar pharmacological properties to curcumin including antioxidant activity (Chen et al., 2006[4]; Somparn et al., 2007[34]), cardiovascular protective effects (Moohammadaree et al., 2015[24]), and anti-inflammatory effects (Zhao et al., 2015[42]). However, the molecular mechanisms associated with the action of HHC on Ang II-induced VSMCs are not well understood. Therefore, this study was designed to explore the mechanisms of action of HHC on the proliferation and migration of VSMCs induced by Ang II via oxidative stress and inflammation.

## Materials and Methods

### Preparation of hexahydrocurcumin (HHC) 

HHC was synthesized from curcumin, which was obtained from *Curcuma longa *rhizomes as described previously (Changtam et al., 2010[3]). In brief, a solution of curcumin in ethanol was hydrogenated by using activated charcoal-supported palladium (Pd/C) as a catalyst for 8 h to yield hydrogenated products, which were then isolated by silica gel column chromatography using dichloromethane-methanol as eluting solvent to afford HHC (85 %) and octahydrocurcumin (10 %). The spectroscopic (^1^H and ^13^C nuclear magnetic resonance (NMR) and mass spectra) data of HHC were consistent with the reported values (Changtam et al., 2010[3]; Jearjaroen et al., 2021[15]). The purity of HHC was more than 97 % from HPLC analysis.

### Cell isolation and cell culture

Male Wistar rats (200-250 g) were supplied by Nomura Siam International Co., Ltd., Pathumwan, Bangkok, Thailand. All animal experimental procedures were approved by the Animal Ethics Committee in accordance with the guidelines for the care and use of laboratory animals published by Chiang Mai University (Protocol Number 19/2565). VSMCs were isolated from the thoracic aorta of rats, as described in a previous study with some modifications (Ikawati et al., 2018[14]). Cells were cultured in Dulbecco's Modified Eagle Medium/Nutrient Mixture F-12 (DMEM-F12) containing 10 % fetal bovine serum (FBS), 100 U/mL penicillin, and 100 U/mL streptomycin at 37 °C in a humidified 5 % CO_2 _incubator. VSMC quiescence was induced by placing cells on serum-free media for 24 h before treatment.

### Cell viability assay

Cell viability was analyzed using the modified tetrazolium salt 3-(4,5-dimethyl-2-thiazolyl)-2,5-diphenyl-2H-tetrazolium bromide (MTT) reduction assay. VSMCs were seeded in 96-well plates at a density 5x10^4 ^cells/ml. The cells were pretreated with HHC (5, 10, 20 and 40 µM) for 2 h and then stimulated with or without Ang II 10 µM (Sigma-Aldrich, MO, USA). The specific inhibitor, namely GKT137831 (10 µM), a NOX1/4 inhibitor was added 1 h before the HHC pretreatment. After 24 h, the medium was removed, and the cells were incubated with MTT (10 mg/ml) at 37 °C for 2 h. Dark blue formazan crystals were solubilized with dimethyl sulfoxide (DMSO) and then the absorbance was measured at 540 nm using a microplate reader (Anthos, Italy).

### Wound healing migration assay

Wound healing assays were used to evaluate the effect of HHC on Ang II mediated VSMC migration. In brief, the cells were seeded into 6-well plates and allowed to rest for 24 h in a serum-free medium. After that, a sterile pipette tip was used to scratch on the underside of the dish to generate a cell-free gap. The cells were washed with phosphate buffer saline (PBS) and pre-treated with a specific dose of HHC (10, 20 and 40 µM) and then stimulated with or without Ang II in a serum-free medium. The images of cell migration across the wound were taken at 0 h and 24 h using an inverted microscope (Carl Zeiss, Goettingen, Germany).

### Transwell migration assay

VSMCs were suspended in a serum-free medium, and the cells at a density of 2x10^5^ cells/ml were seeded in the upper chamber of a 24-well plate transwell with a polycarbonate membrane, 8 µM pore size (Corning, Maine, USA). Media with or without Ang-II were added into the lower chamber. Then, the cells were pretreated with HHC (10, 20 and 40 µM) for 2 h. The specific inhibitor namely GKT137831 (10 µM) was added 1 h before the HHC pretreatment. After incubating for 24 h, the non-migrated cells in the upper chamber were removed by cotton swabs. The migrated cells were fixed with 4 % paraformaldehyde and stained with 0.1 % crystal Violet (Sigma-Aldrich, MO, USA). After rinsing with PBS, the stained cells were observed and photographed. The numbers of migrated cells were counted.

### Intracellular ROS assay

Dichlorofluorescin diacetate (DCFH-DA) was used to determine the effect of HHC on intracellular ROS generated in Ang II induced VSMCs. In brief, the confluent cells in the 96-well plates at 5x10^4^ cells/ml were treated with HHC (10, 20 and 40 µM) for 2 h followed by incubation with Ang II (10 µM) for 24 h. Prior to ROS detection, the medium was removed, and the cells were washed with 1X PBS and incubated with 20 µM of DCFH-DA at 37 °C for 30 min. The fluorescence intensity of intracellular ROS production was evaluated at an excitation and emission wavelength of 485 and 530 nm, respectively, using a microplate reader (BioTek Instrument, Winooski, VT, USA).

### Western blot analysis

After treatment, the cells were rinsed with 1X PBS and lysed with ice-cold radioimmunoprecipitation assay (RIPA) lysis buffer with protease inhibitor for total proteins. For investigation of the cytoplasmic proteins, the cells were added to ice-cold hypotonic lysis buffer (10 mM HEPES, pH 7.9; 1.5 mM MaCl_2_; 10 mM KCl; 0.5 PMSF; 0.5 mM DTT) and protease inhibitor at 4 °C for 15 min and centrifuged at 13,000 r/min. The nuclear pellets were resuspended in ice-cold hypertonic buffer (10 mM HEPES, pH 7.9; 0.42 M NaCl; 1.5 mM MgCl_2_; 10 mM KCl; 0.5 mM PMSF; 1mM DTT) and protease inhibitors at 4 °C for 40 min and centrifuged at 4 °C for 10 min. The protein concentrations were measured using Bradford protein assay (Bio-Rad Laboratories, Hercules, CA, USA). Approximately 25 µg of protein extract was separated by 10-15 % sodium dodecyl sulfate-polyacrylamide gel electrophoresis (SDS-PAGE) and transferred to polyvinylidene difluoride (PVDF) membranes. The membranes were blocked with 5 % skim-milk in Tris-buffered saline (TBS) containing 0.1 % Tween 20 (TBST) buffer at room temperature for 2 h and then incubated with primary antibodies recognizing NOX1 (1:1000, Abcam), NOX4 (1:1000, Santa Cruz Biotechnology), PPAR-γ (1:1000, Abcam), PGC-1α (1:1000, ThermoFisher Scientific), Cyclin D1 (1:1000, Cell Signaling Technology), p21 (1:1000, Santa Cruz Biotechnology), NF- κB (1:1000, Cell Signaling Technology), TNF-α (1:1000, Abcam), IL-6 (1:1000, Abcam), MMP9 (1:1000, Merck Darmstadt, Germany), Lamin B (1:1000, Affinity) or β-actin (1:20000, Cell Signaling Technology) overnight at 4 °C. After incubation with horseradish peroxidase-conjugated secondary antibodies for 2 h, the protein bands were visualized with the enhanced chemiluminescence detection system (Aplegen Gel Company, Inc., San Francisco, CA, USA).

### Statistical analysis

Data are expressed as the mean ± standard error of mean (SEM). Comparisons of more than two groups were carried out using a one-way ANOVA followed by Dunnett's test using GraphPad Prism software. The differences were considered statistically significant at p<0.05.

## Results

### Effects of HHC on Ang II-induced cell proliferation in VSMCs

**Figure 2:**
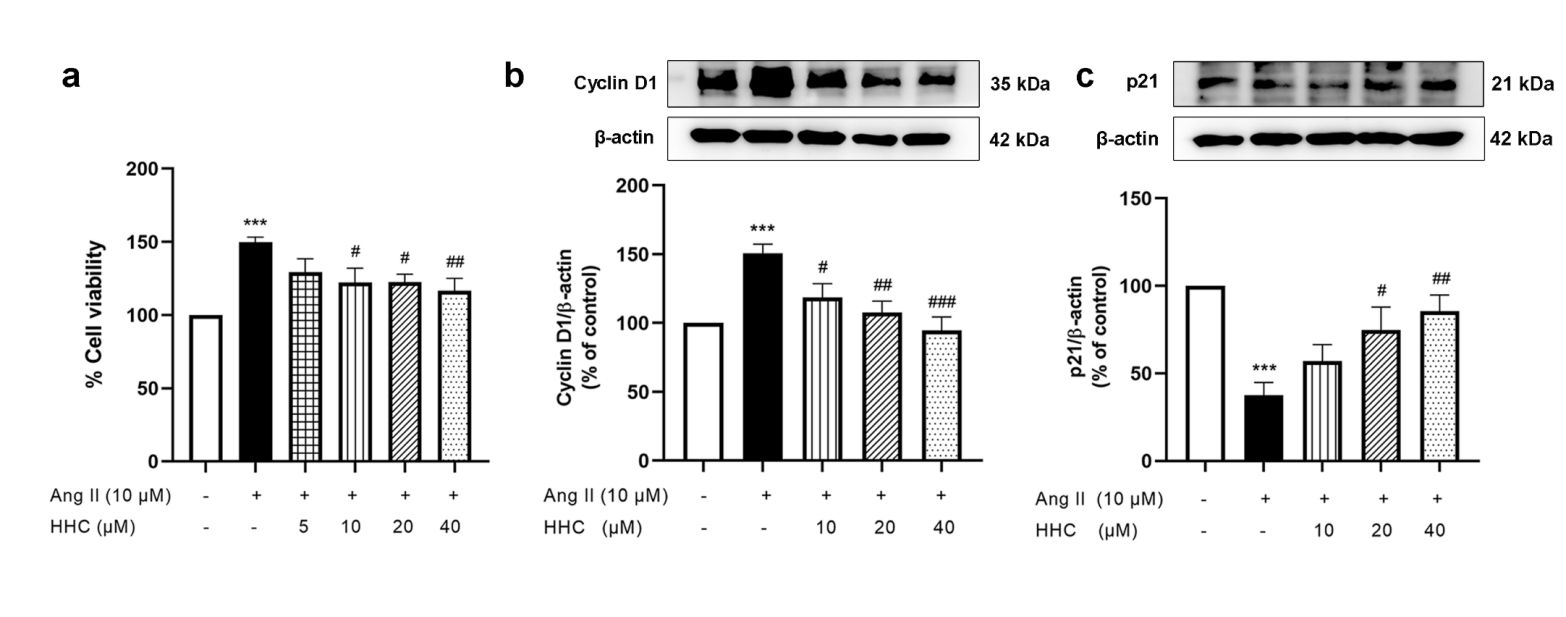
Effects of HHC on Ang II-induced VSMCs proliferation. (a) HHC attenuated Ang II-induced proliferation of VSMCs a in dose-dependent manner; (b-c) Western blot data showing the effect of HHC on the expression of cyclin D1 and p21 in VSMCs induced by Ang II. Data are expressed as mean ± SEM (n=6). ***, p<0.001, versus control group; ^#^, p<0.05; ^##^, p<0.01; and ^###^, p<0.001, versus Ang II group

To investigate the effect of HHC on the proliferation of VSMCs cells were pretreated with HHC (5, 10, 20 and 40 µM) before being exposed to Ang II (10 µM) for 24 h. The results showed that Ang II stimulated VSMC proliferation statistically significantly when compared to the control group (p<0.001) (Figure 2a(Fig. 2)). Pretreatment of VSMCs with HHC (10, 20 and 40 µM) dose-dependently attenuated the proliferation of the cells (Figure 2a(Fig. 2)). Cell proliferation is regulated in the cell cycle by sequential activation and inactivation of cyclin-dependent kinases (CDKs), a family of serine/threonine protein kinases (Huang et al., 2010[12]; Karimian et al., 2016[18]). The expressions of the regulatory proteins of the cell cycle including cyclin D1 and the CDK inhibitor p21 were investigated by western blotting. The results showed that Ang II-induction of VSMCs significantly increased the expression protein of cyclin D1 (Figure 2b(Fig. 2)), while the expression protein of p21 decreased (Figure 2c(Fig. 2)). Pretreatment with HHC diminished the expression of cyclin D1 and increased the expression of p21 in Ang II-induced VSMCs as shown in Figure 2b-c(Fig. 2). Overall, these results suggested that HHC could inhibit Ang II-induced VSMC proliferation by suppressing the expression of cyclin D1 and increasing that of p21.

### Effects of HHC on Ang II-induced cell migration in VSMCs

**Figure 3:**
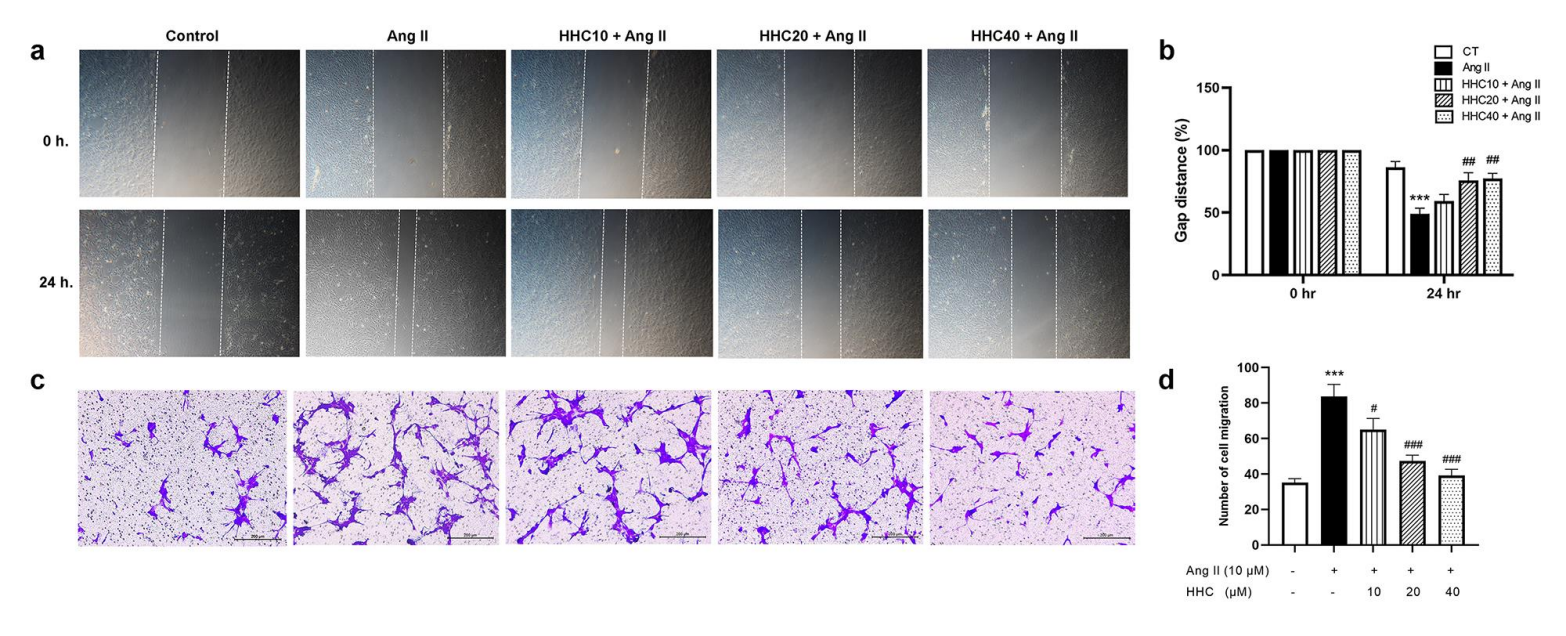
Effects of HHC on Ang II-induced VSMCs migration. (a) Imaging from wound healing assay and (b) gap distance as measured by ImageJ software. (c) Cell migration was evaluated using the transwell Boyden chamber assay. (d) The number of migrate cells was counted and analyzed statistically. Data are expressed as mean ± SEM (n=6). ***, p<0.001, versus control group; ^#^, p<0.05; ^##^, p<0.01; and ^##^, p<0.001, versus Ang II group

To determine the effects of HHC on Ang II-induced VSMC migration, a wound-healing assay and transwell Boyden chamber assay were carried out. The results showed that Ang II significantly increased the migration rate (p<0.001) (Figure 3a-b(Fig. 3)) and the transwell Boyden chamber assay also revealed that the number of cells migrating markedly increased in Ang II-induced VSMCs compared to the control group (Figure 3c-d(Fig. 3)). Following pretreatment with HHC before Ang II treatment, the migration rate of VSMCs and the number of VSMCs migrating were observed to decrease significantly compared with that of the group treated with Ang II alone (Figure 3(Fig. 3)). These results indicate that HHC could inhibit the VSMC migration.

### Effects of HHC on Ang II-induced ROS generation and NADPH oxidase expression in VSMCs

**Figure 4:**
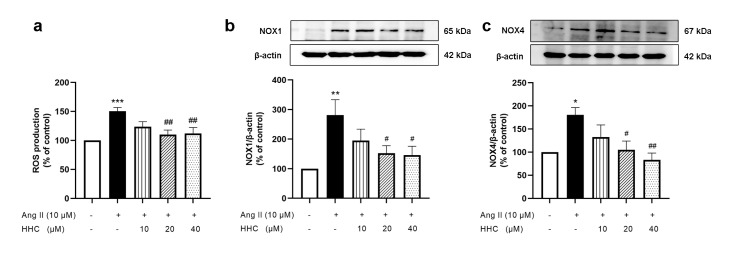
Effects of HHC on Ang II-induced ROS production and expression of NADPH oxidase in VSMCs. (a) ROS production was investigated by DCFH-DA assay. (b-c) The effect of HHC on the expression of NOX1 and NOX4 protein, as investigated by western blot analysis. Data are expressed as mean ± SEM (n=6). *, p<0.05; **, p<0.01; and ***, p<0.001, versus control group; ^#^, p<0.05; and ^##^, p<0.01, versus Ang II group

Ang II is a powerful activator of oxidative stress which can trigger multiple pathological responses including proliferation, migration of VSMCs and overexpression of proinflammatory cytokines (Nguyen Dinh Cat et al., 2013[25]). To investigate the effects of HHC on Ang II-induced ROS generation in VSMCs, intracellular ROS levels were evaluated by DCFH-DA assay. The results showed that ROS levels significantly increased in Ang II-induced VSMCs compared to the control group (p<0.001) (Figure 4a(Fig. 4)). Pretreatment with HHC reduced ROS levels in Ang II-induced VSMCs (Figure 4a(Fig. 4)). NOXs are recognized as the major source to produce ROS in VSMCs. The expression of the proteins NOX1 and NOX4 were investigated by western blotting. Treatment with Ang II resulted in a significant increase in the expression of NOX1 (Figure 4b(Fig. 4)) and NOX4 (Figure 4c(Fig. 4)) in comparison with that of the control group. Following pretreatment with HHC before Ang II treatment, the expression of NOX1 and NOX4 was observed to decrease significantly compared with that of the group treated with Ang II alone (Figure 4b-c(Fig. 4)). These results indicate that HHC attenuates Ang II-induced ROS generation by inhibiting NADPH oxidases.

### Effects of HHC on Ang II-induced inflammation in VSMCs

**Figure 5:**
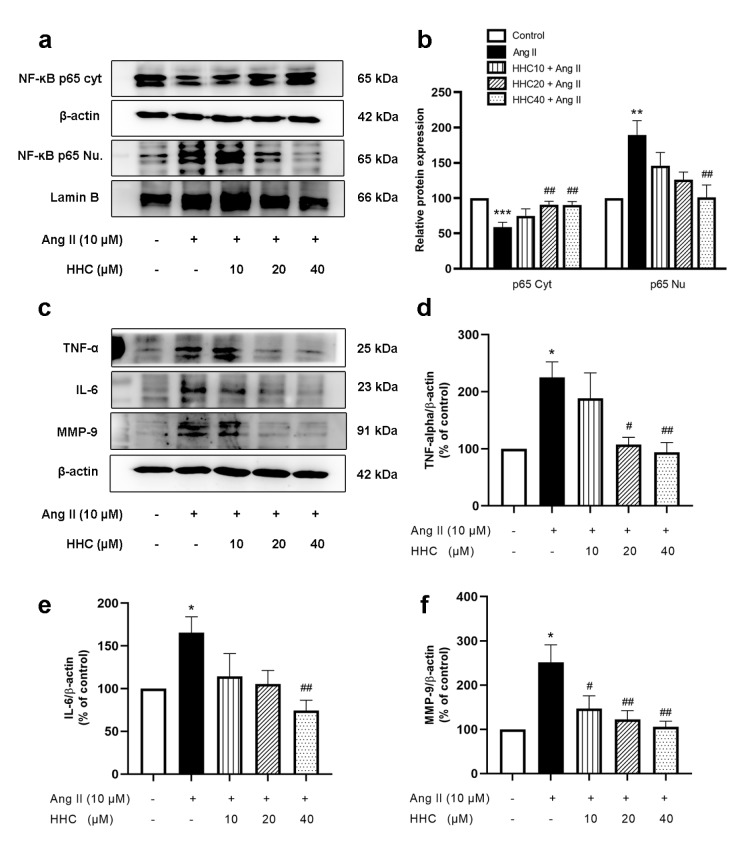
Effects of HHC on Ang II-induced inflammation in VSMCs. Cells were pretreated with HHC for 2 h before incubation in the presence or absence of Ang II for 24 h. (a) Representative bands of the expression of NF-кB p65 in cytosol and nucleus, as investigated by western blot analysis. (b) The quantitative results of the expression of NF-кB p65 in cytosol and nucleus in each group. (c) Representative bands of the TNF-α, IL-6 and MMP9 protein expression, as investigated by western blot analysis. (d-f) The quantitative results of TNF-α, IL-6 and MMP9 protein expression. Data are expressed as mean ± SEM (n=6). *, p<0.05; **, p<0.01; and ***, p<0.001, versus control group; ^#^, p<0.05; and ^##^, p<0.01, versus Ang II group

Ang II activates the redox-sensitive proinflammatory transcription factor, nuclear factor (NF)- κB, which is associated with stimulation of cytokine production (Touyz, 2003[37]). To explore the mechanism underlying the effect of HHC in causing the reduction of Ang II-induced inflammation in VSMCs mediated by the NF- κB pathway, the expression of nuclear NF- κB p65 and cytosolic NF- κB p65 and inflammatory cytokines were assessed by western blotting. As shown in Figure 5a-b(Fig. 5), the expression of NF κB p65 was mainly observed in the nucleus, whilst the level of the cytosolic NF κB p65 was significantly decreased in Ang II-induced VSMCs. At the same time, the expression of TNF-α (Figure 5c-d(Fig. 5)) and IL-6 (Figure 5c, e(Fig. 5)) was also found to have increased compared with that of the control group. Following pretreatment with HHC before Ang II treatment, a reduction of the translocation of NF- κB p65 was observed, which is closely related to the finding that the expression of the proteins TNF-α and IL-6 were also significantly reduced (Figure 5c-e(Fig. 5)). ROS and inflammatory cytokines also stimulate the expression of MMPs which are required for VSMC migration. Therefore, the expression of the protein MMP9 was evaluated by western blotting. The results showed that the expression of MMP9 was significantly enhanced in VSMCs treated with Ang II in comparison to the control cell group (p<0.05) and decreased in VSMCs pretreated with HHC before exposure to Ang II (Figure 5c, f(Fig. 5)). These results suggest that HHC diminished the activation of NF- κB, the activation of TNF-α , IL-6 and the expression of MMP9 in Ang II-induced VSMCs.

### Effects of HHC on Ang II-inhibited PPAR-γ and PGC-1α expression in VSMCs

**Figure 6:**
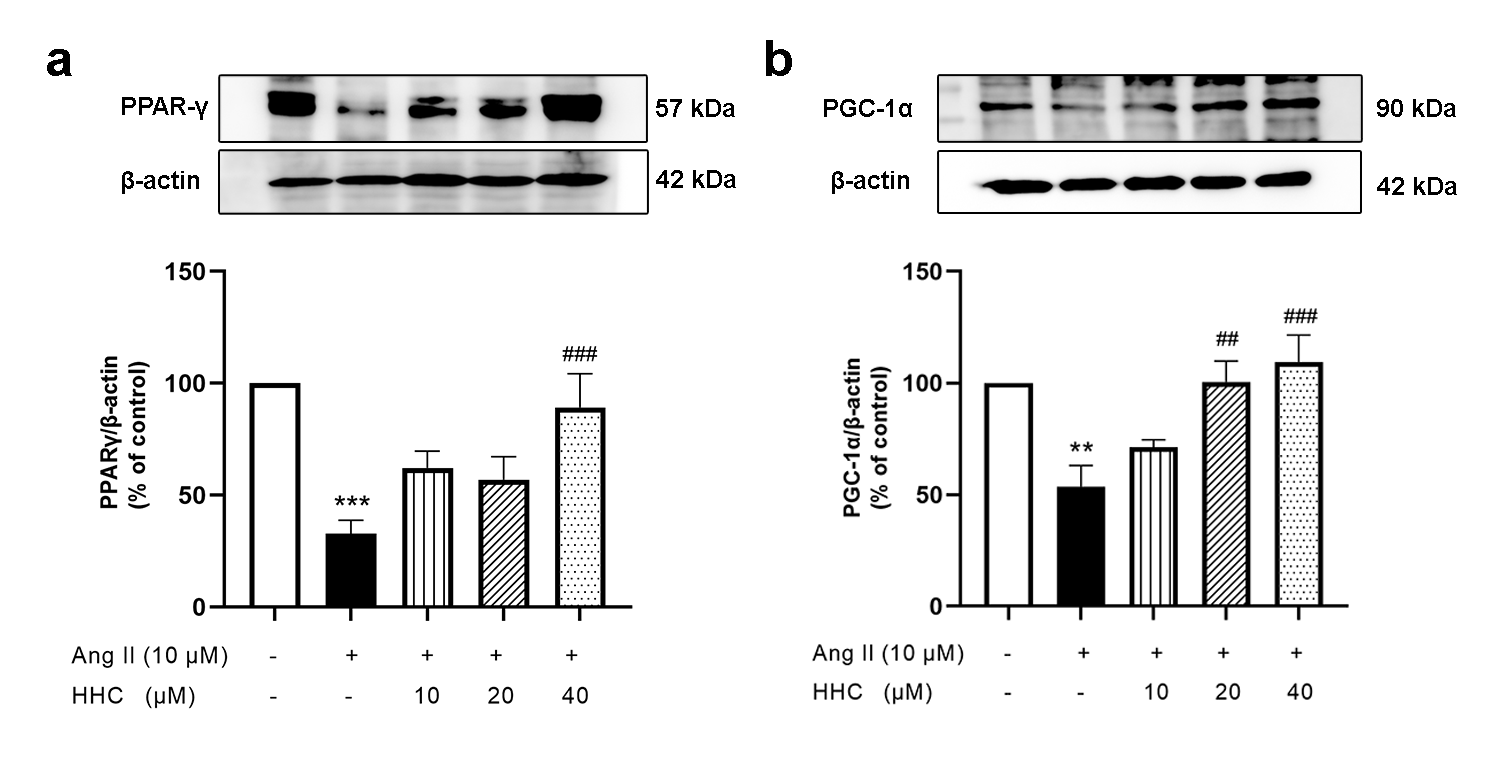
Effects of HHC on Ang II-suppressed expression of PPAR-γ and PGC-1α in VSMCs. The expression of PPAR-γ (a) and PGC-1α (b) was detected by western blot analysis. Data are expressed as mean ± SEM (n=6). **, p<0.01; and ***, p<0.001, versus control group; ^##^, p<0.01; and ^###^, p<0.001, versus Ang II group

PPAR-γ is present in VSMCs in the normal vascular wall and it can suppress the expression of inflammatory genes (Duan et al., 2008[9]). It has been reported that PGC-1α is a powerful regulator of ROS metabolism (Pei et al., 2016[29]) and also decreases the activity of NF- κB in vasculature (Kadlec et al., 2016[17]). To determine whether HHC could enhance the expression of PPAR-γ and PGC-1α in Ang II-induced VSMCs western blot analysis was used to examine the expression of the proteins PPAR-γ and PGC-1α. As shown in Figure 6(Fig. 6), the expression of PPAR-γ (Figure 6a(Fig. 6)) and PGC-1α (Figure 6b(Fig. 6)) were suppressed in Ang II-induced VSMCs and HHC pretreatment significantly restored the expression of PPAR-γ and PGC-1α in Ang II-induced VSMCs. These results indicate that PPAR-γ and PGC-1α are required for HHC to reverse the proliferation, migration and inflammation induced by Ang II.

### Effects of HHC on Ang II-induced VSMC proliferation, migration and inflammation involved generation of NADPH oxidase-mediated ROS

**Figure 7:**
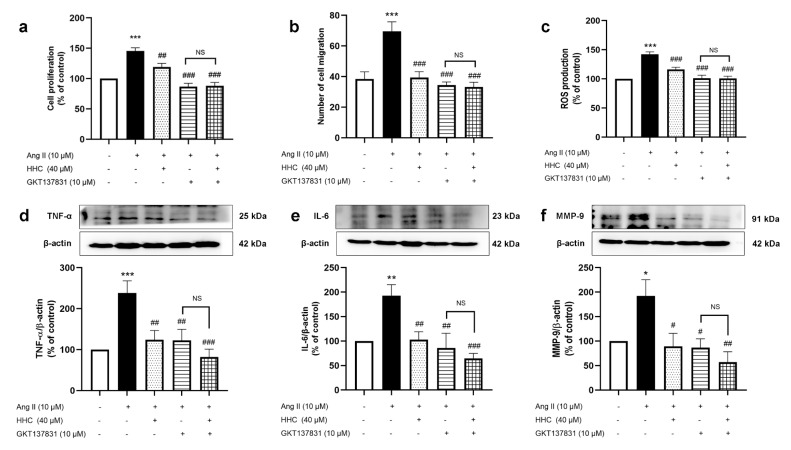
The inhibitory effects of HHC on Ang II-induced VSMCs proliferation, migration and inflammation mediated by the inhibition of NADPH oxidase-mediated ROS generation. VSMCs were pretreated with HHC (40 µM), GKT137831 (10 µM) or their combination before being exposed to Ang II for 24 h. (a) Showing cell proliferation from MTT assay. (b) Data from transwell assay to show the number of migrated cells. (c) ROS levels as detected by DCFH-DA assay. (d-f) The expression of TNF-α, IL-6 and MMP9 protein as detected by western blot analysis. Data are expressed as mean ± SEM (n=6). *, p<0.05; **, p<0.01; and ***, p<0.001, versus control group; ^#^, p<0.05; ^##^, p<0.01; and ^###^, p<0.001, versus Ang II group. NS indicates no significance.

To clarify the mechanics of the protective effect of HHC on Ang II-induced cell proliferation, migration, and inflammation by determining whether it acts related with NADPH oxidase-mediated ROS production, we investigated the impact of GKT137831, a NOX1/4 inhibitor in this study. As shown in Figure 7a-b(Fig. 7), treatment with Ang II increased the proliferation and migration of VSMCs, whereas pretreatment with HHC was found to decrease these actions. Moreover, treatment with HHC and GKT137831 showed similar patterns to the HHC pretreatment. In addition, ROS levels (Figure 7c(Fig. 7)) and the expression of TNF-α (Figure 7d(Fig. 7)), IL-6 (Figure 7e(Fig. 7)), and MMP9 (Figure 7f(Fig. 7)) also decreased when the cells were pretreated with HHC and GKT137831. These results suggest that HHC attenuated Ang II-induced VSMC proliferation, migration and inflammation mediated by NOX1/4 mediated ROS generation. 

See also Supplementary data and Supplementary data western blots.

## Discussion and Conclusion

HHC has been shown to impact multiple biological activities in both *in vivo* and *in vitro* studies such as antioxidant, anti-inflammatory, anticancer and cardiovascular protective effects (Huang et al., 2018[13]). In addition, a previous study showed that HHC also attenuated high blood pressure and vascular remodeling in L-NAME induced rats (Panthiya et al., 2022[28]). On considering what is already believed, this study mainly focused on the effects of HHC on cell proliferation, migration, and inflammation in Ang II-induced VSMCs and the underlying mechanisms associated with these processes.

Angiotensin II (Ang II) is the primary effector molecule of the renin angiotensin system (RAS) (Mehta and Griendling, 2007[23]). It is well known that Ang II stimulates the proliferation, migration, and inflammation of VSMCs and induces oxidative stress all of which play vital roles in the pathogenesis of atherosclerosis, hypertension, and other cardiovascular diseases (Pei et al., 2016[29]; Shen et al., 2014[33]). Therefore, inhibition of Ang II-induced VSMC proliferation and migration may serve as a potential therapeutic strategy for such diseases. This study found that HHC inhibits Ang II-induced VSMC proliferation and migration in a dose-dependent manner**.** Cell proliferation occurs when resting cells are stimulated to enter the cell cycle by a growth factor (Berridge, 2014[2]). Cyclin D1, a regulatory protein of the cell cycle that controls G_1_ progression is the main target of the growth factor signaling pathway. p21, a CDK inhibitor plays an important role in arresting the cell cycle in the G_1_ phase in response to a variety of stimuli. Failure to arrest the cell cycle resulting from loss of the CKI function leads to excessive proliferation (Duronio and Xiong, 2013[10]). Previous studies reported that Ang II upregulated cyclin D1 and decreased the expression of p21 in VSMCs leading to progression of the cell cycle and cell proliferation (Pantan et al., 2016[27]; Pei et al., 2016[29]). Moreover, p21 also plays a role in the suppression of the migration of VSMCs (Pantan et al., 2016[27]). This study found that HHC downregulated cyclin D1 and enhanced the expression of p21 in Ang II-induced VSMCs. 

There are several reports demonstrating the key effects Ang II plays in vascular injury, mediated by induction of the NADPH oxidase signaling pathway and the NF- κB signaling pathway. Ang II stimulates NADPH oxidase to produce ROS that modulate many downstream signaling molecules leading to the growth and migration of VSMCs, expression of pro-inflammatory mediators and modification of the extracellular matrix (Touyz, 2004[36]). NOX1 and NOX4 are the predominant isoforms of NOX in the VSMCs of rodents (Clempus and Griendling, 2006[6]; Schroder, 2010[32]). Previous studies reported that in response to Ang II, over-expression of NOX1 and increased ROS production were significantly observed in the VSMCs of transgenic mice (Dikalova et al., 2010[8]). Ang II enhanced ROS production, NADPH oxidase activation and the expression of NOX4 in VSMCs (Zhang et al., 2019[41]). A previous report stated that HHC eliminated free radicals and possessed antioxidant effects in an *in vitro* study (Huang et al., 2018[13]). In addition, HHC was shown to diminish oxidative stress and increase antioxidative enzymes in cerebral ischemia/reperfusion rats (Wicha et al., 2017[39]). Our study found that pretreatment with HHC attenuated ROS production and the expression of NOX1 and NOX4 in VSMCs induced by Ang II. Ang II and ROS can activate transcription factors including NF- κB, which, in turn, leads to activation of transcription of pro-inflammatory cytokines such as TNF-α and IL-6, adhesion molecules including MMPs leading to cell proliferation and migration (Dandona et al., 2007[7]; Ruiz-Ortega et al., 2002[31]). Moreover, NF- κB can directly regulate cyclin D in the cell cycle resulting in cell growth (Duronio and Xiong, 2013[10]). Inhibition of the activation of NF- κB can suppress the proliferation of human VSMCs (Wang et al., 2002[38]). HHC could suppress the activation of NF- κB in RAW 264.7 macrophages induced by LPS (Pan et al., 2000[26]). In this study, HHC inhibited the translocation of NF- κB into the nuclei resulting in a decrease in the expression of the proteins TNF-α , IL-6 and MMP9. Furthermore, this study investigated whether the inhibitory effects of HHC on Ang II-induced cell proliferation, migration and inflammation are related to ROS mediated by NOX1/4 by using GKT137831 (a NOX1/4 inhibitor). The results indicated that HHC and GKT137831 abolished the effects induced by Ang II. In this study, it was found that inhibition of NOX1/4 led to a reduction in oxidative stress, and overall inflammatory response resulting in a decrease in cell proliferation and migration in VSMCs induced by Ang II. 

There are several reports demonstrating the anti-inflammatory effect of PPAR-γ, a member of the nuclear hormone receptor superfamily of ligands, which plays an important role in regulating energy metabolism (Li et al., 2017[19]; Marchesi et al., 2013[21]). Rosiglitazone, a PPAR-γ agonist has been shown to suppress Ang II-induced inflammation in *in vivo* and *in vitro* studies (Ji et al., 2009[16]). Moreover, the activation of PPAR-γ could attenuate Ang II-induced cell growth, proliferation, and inflammation responses in VSMCs (Ji et al., 2009[16]; Marchesi et al., 2013[21]). The present results support the findings of previous studies that Ang II promotes proliferation and inflammation in VSMCs. Our results found that HHC could enhance the expression of PPAR-γ as well as suppress the inflammatory response in VSMCs. Additionally, PGC-1α is a transcriptional coactivator linked to energy metabolism and antioxidant defense. Overexpression of PGC-1α can reduce ROS-mediated VSMCs migration (Qu et al., 2009[30]) and proliferation (Kadlec et al., 2016[17]). It has been shown that PGC-1α inhibited proliferation and migration, ROS production and NADPH oxidase activation in Ang II-induced VSMCs (Zhao et al., 2015[43]). Our study found that HHC could enhance the expression of PGC-1α in VSMCs induced by Ang II. These results indicated that the inhibitory effects of HHC on VSMC proliferation and migration may be related to suppression of PPAR-γ and PGC-1α.

In conclusion, our findings demonstrate that HHC has a potent suppressive effect on proliferation, migration, and inflammation in VSMCs. The inhibitory effects of HHC may partially depend on suppression of ROS generation mediated by NOX1/4 and the increase in expression of the proteins PPAR-γ and PGC-1α. Our findings suggest that HHC may be a potential therapeutic agent and the findings support further investigation into the role of HHC in the prevention and treatment of vascular diseases.

## Declaration

### Conflict of interest

The authors declare no conflict of interest.

### Acknowledgments

This study was supported by the Thailand Science Research and Innovation (TSRI) fund, Thailand, Center of Excellence for Innovation in Chemistry, Ministry of Higher Education, and the Royal Golden Jubilee (RGJ) PhD program (Grant No. PHD/0107/2561) by the National Research Council of Thailand (NRCT).

## Supplementary Material

Supplementary data

Supplementary data western blots

## Figures and Tables

**Figure 1:**
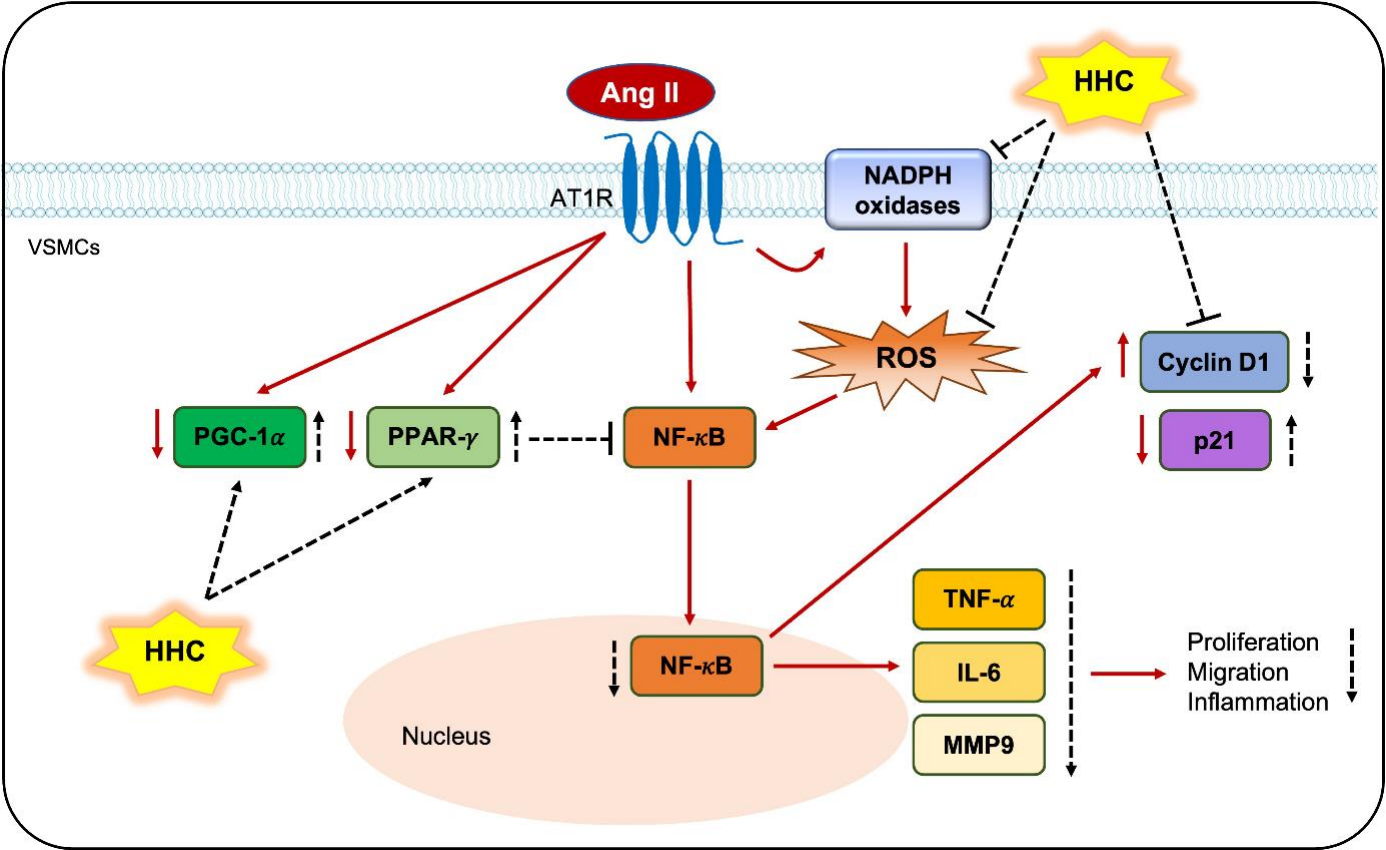
Graphical abstract
