# Supplementary material for: Hexahydrocurcumin mitigates angiotensin II-induced proliferation, migration, and inflammation in vascular smooth muscle cells
Source: EXCLI J. 2023 Jun 5;22:466–81. doi: 10.17179/excli2023-6124 (PMC10391613; doi:10.17179/excli2023-6124)

EXCLI Journal 2023;22:466-481 – ISSN 1611-2156
Received: April 23, 2023, accepted: May 30, 2023, published: June 05, 2023

Raw data from Western blotting to:

Original article:

HEXAHYDROCURCUMIN MITIGATES ANGIOTENSIN II-INDUCED PROLIFERATION, MIGRATION, AND INFLAMMATION IN VASCULAR SMOOTH MUSCLE CELLS

Luckika Panthiya[1,5], Jiraporn Tocharus[2], Waraluck Chaichompoo[3], Apichart Suksamrarn[3], Chainarong Tocharus[1,4,*]

1 Department of Anatomy, Faculty of Medicine, Chiang Mai University, Chiang Mai 50200, Thailand
2 Department of Physiology, Faculty of Medicine, Chiang Mai University, Chiang Mai 50200, Thailand
3 Department of Chemistry and Center of Excellence of Innovation in Chemistry, Faculty of Science, Ramkhamhaeng University, Bangkok 10240, Thailand
4 Functional Food Research Center for Well-Being, Chiang Mai University, Chiang Mai 50200, Thailand
5 Graduate School, Chiang Mai University, Chiang Mai 50200, Thailand

* **Corresponding author:** Chainarong Tocharus, Department of Anatomy, Faculty of Medicine, Chiang Mai University, Chiang Mai 50200, Thailand. Tel.: +66 53 945312, Fax: +66 53 945304, E-mail: chainarongt@hotmail.com

https://dx.doi.org/10.17179/excli2023-6124

This is an Open Access article distributed under the terms of the Creative Commons Attribution License (http://creativecommons.org/licenses/by/4.0/).

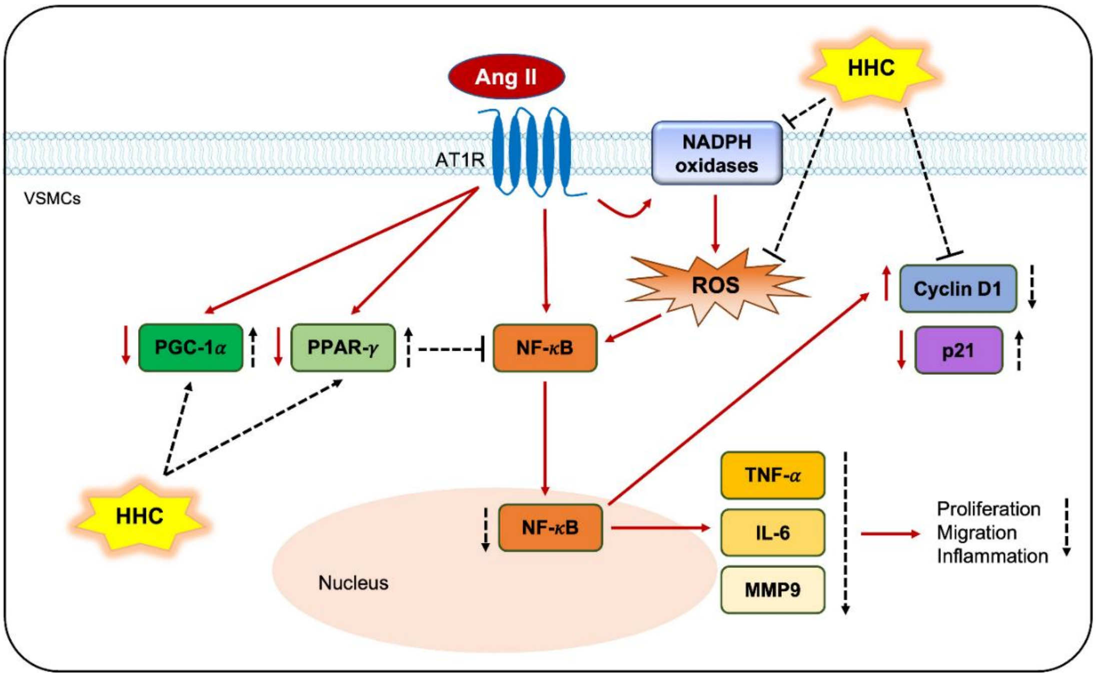

Figure 1: Graphical abstract

Raw data from Western blotting

Cyclin D1 (35 kDa)

In figure 2

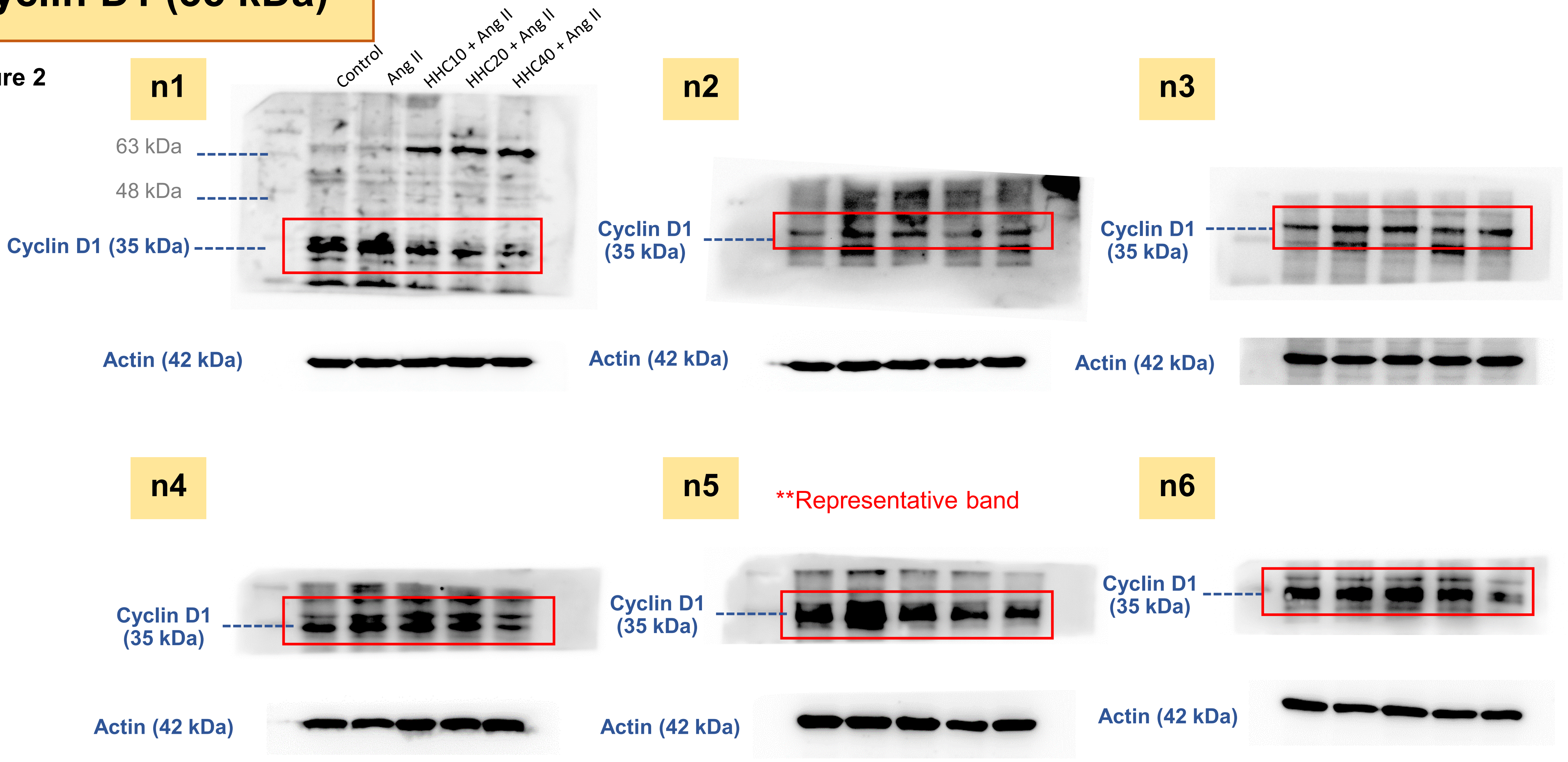

P21 (21 kDa)

In figure 2

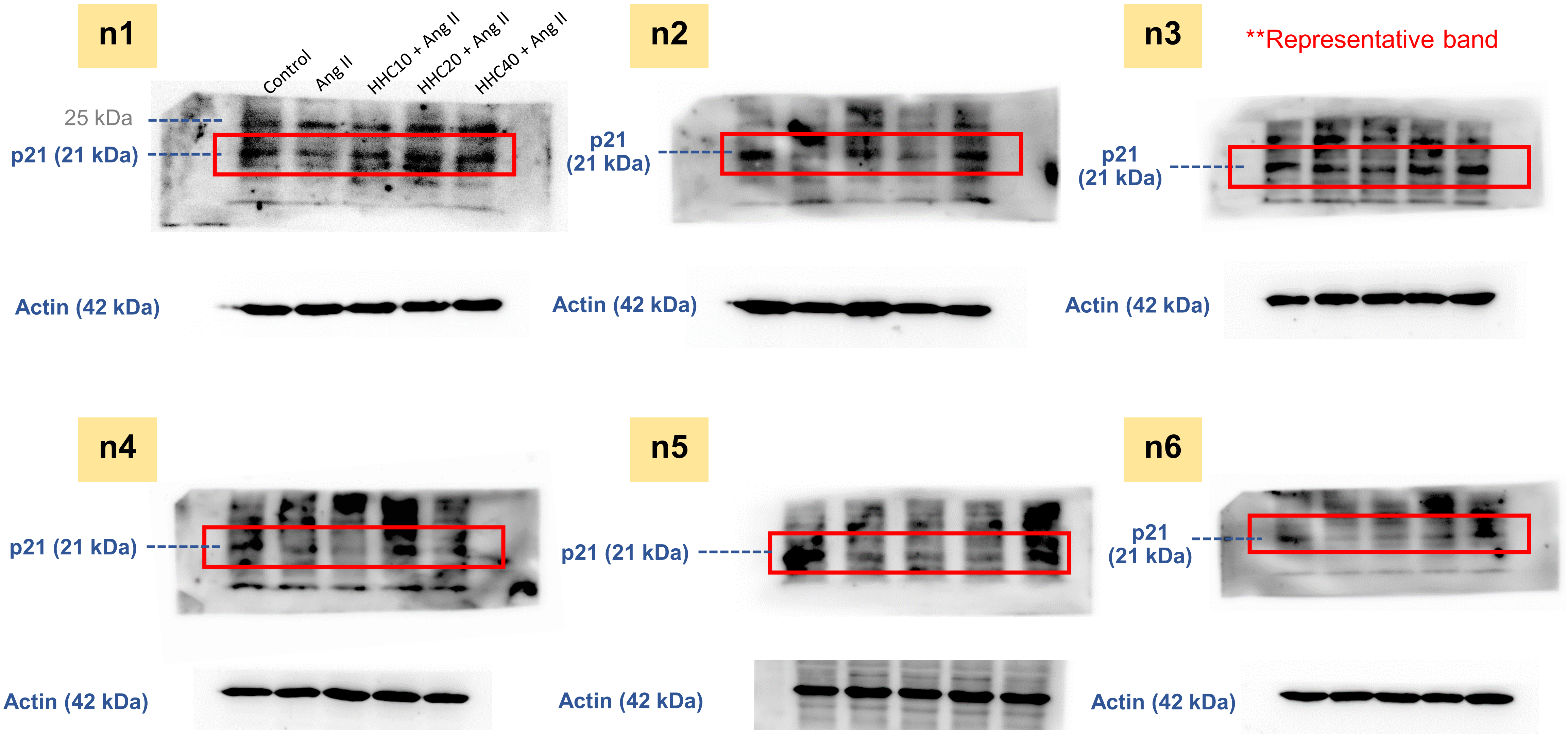

NOX1 (65 kDa)

In figure 4

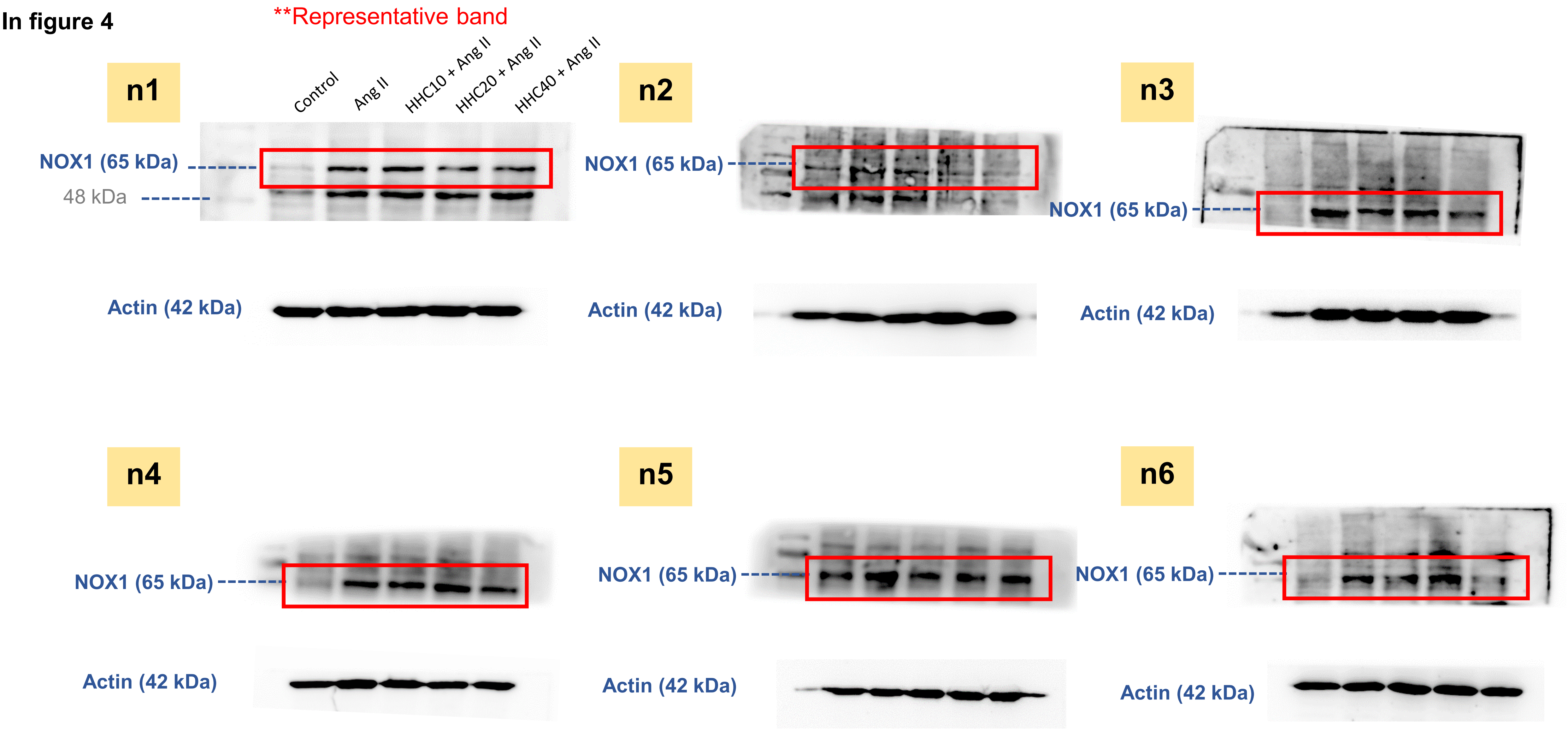

NOX4 (67 kDa)

In figure 4

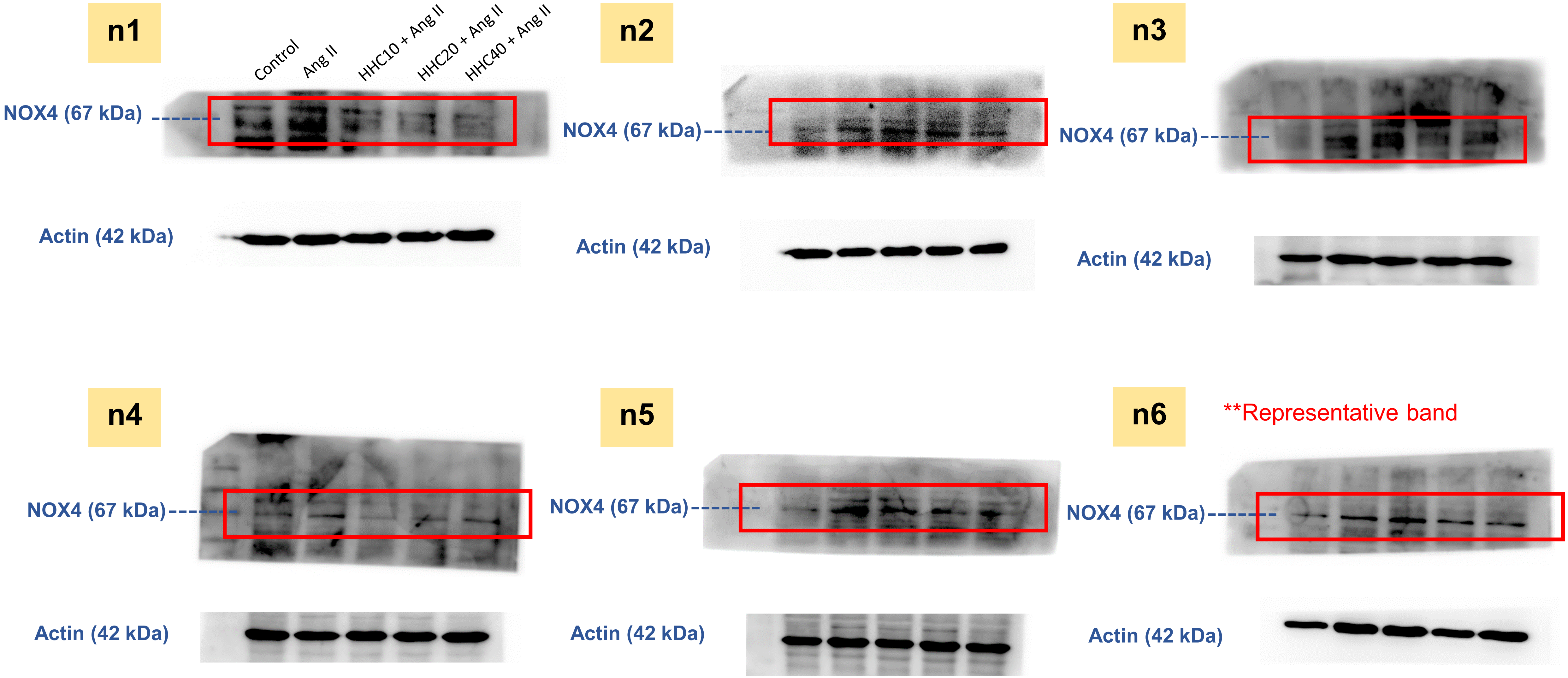

Cytosolic NF-kB p65 (65 kDa)

In figure 5

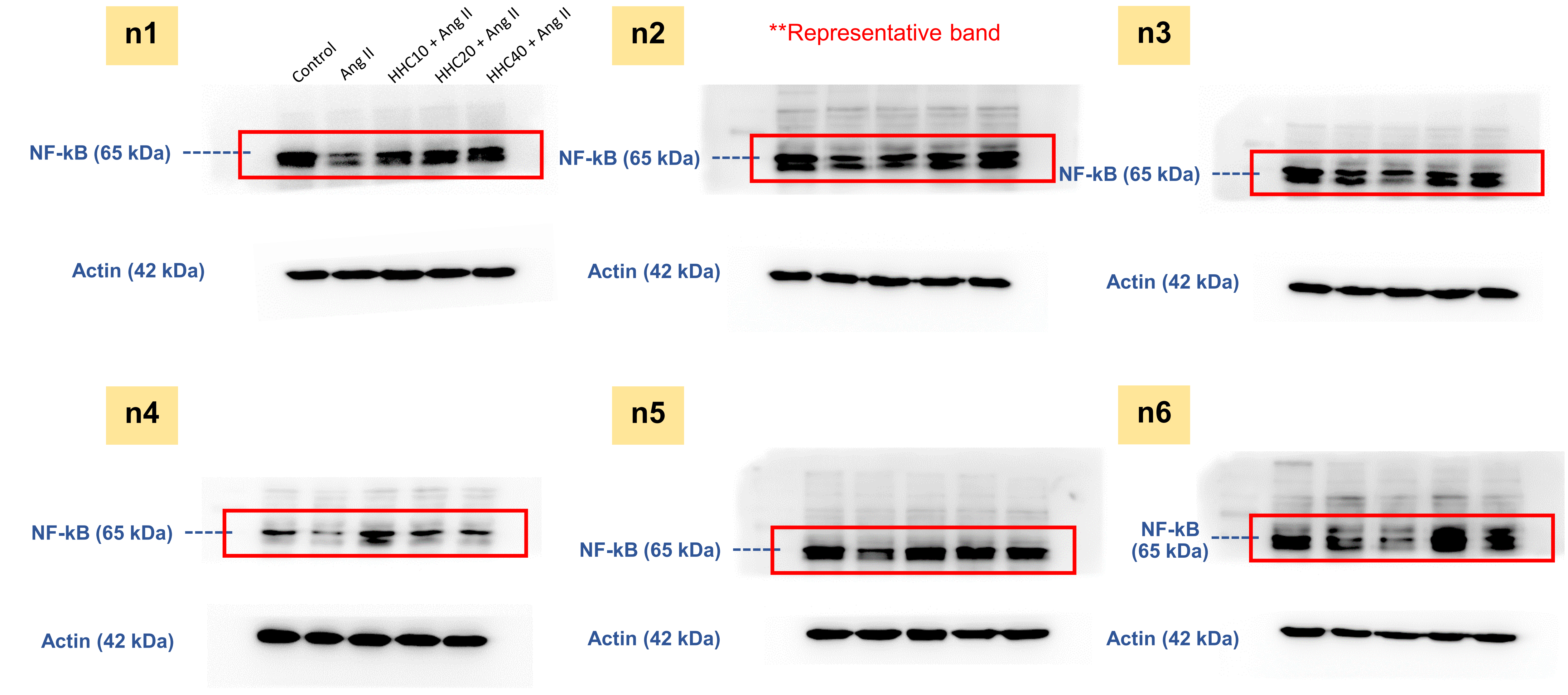

Nuclear NF-kB p65 (65 kDa)

In figure 5

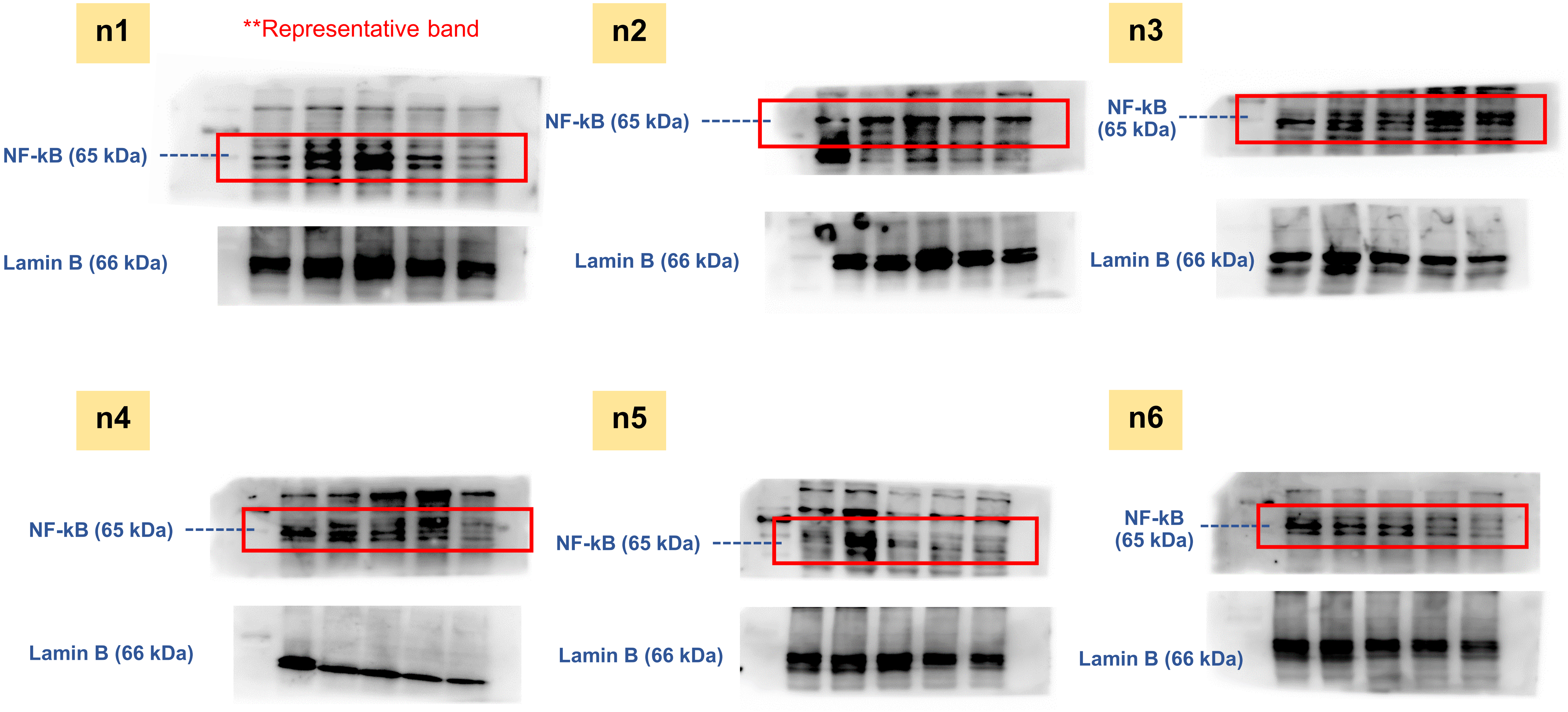

TNF-α (25 kDa)

In figure 5

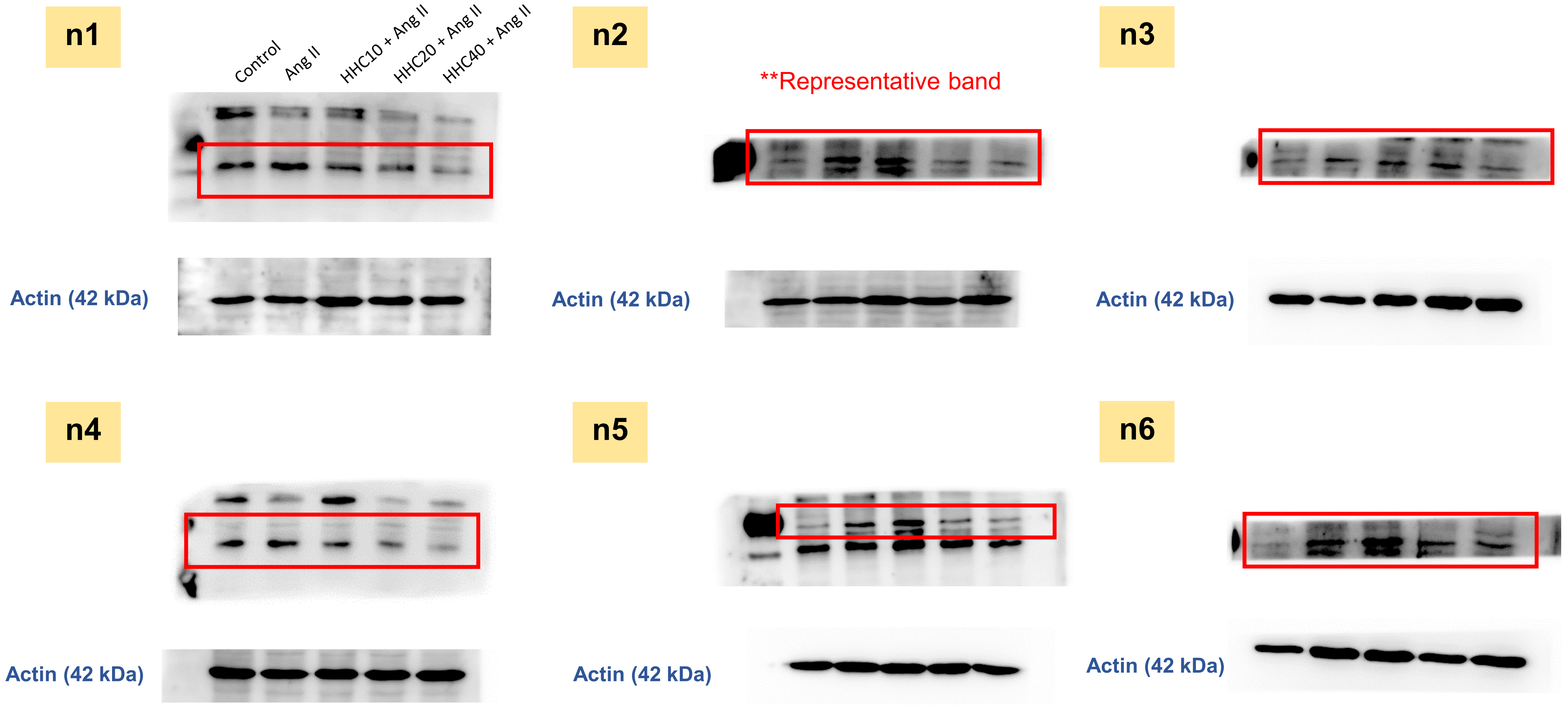

IL-6 (23 kDa)

In figure 5

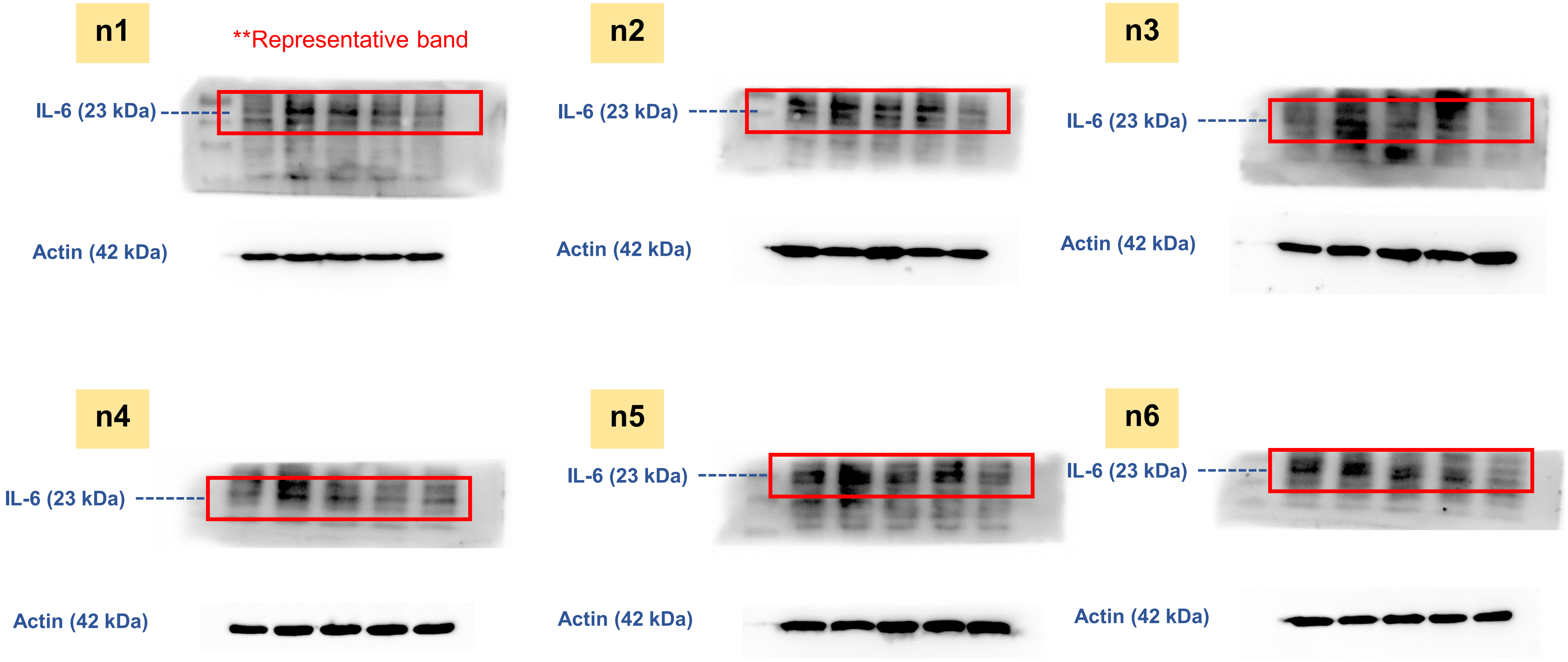

MMP9 (91 kDa)

In figure 5

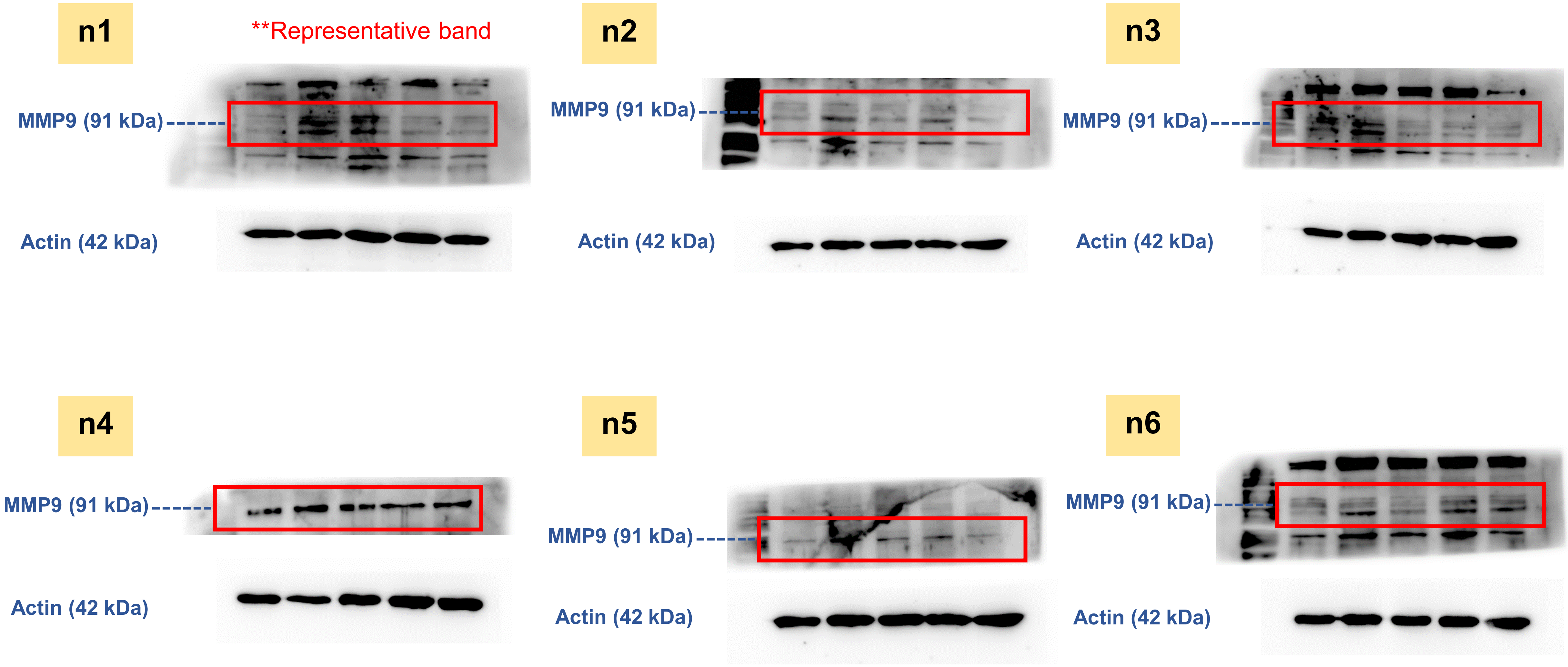

PPARγ (57 kDa)

In figure 6

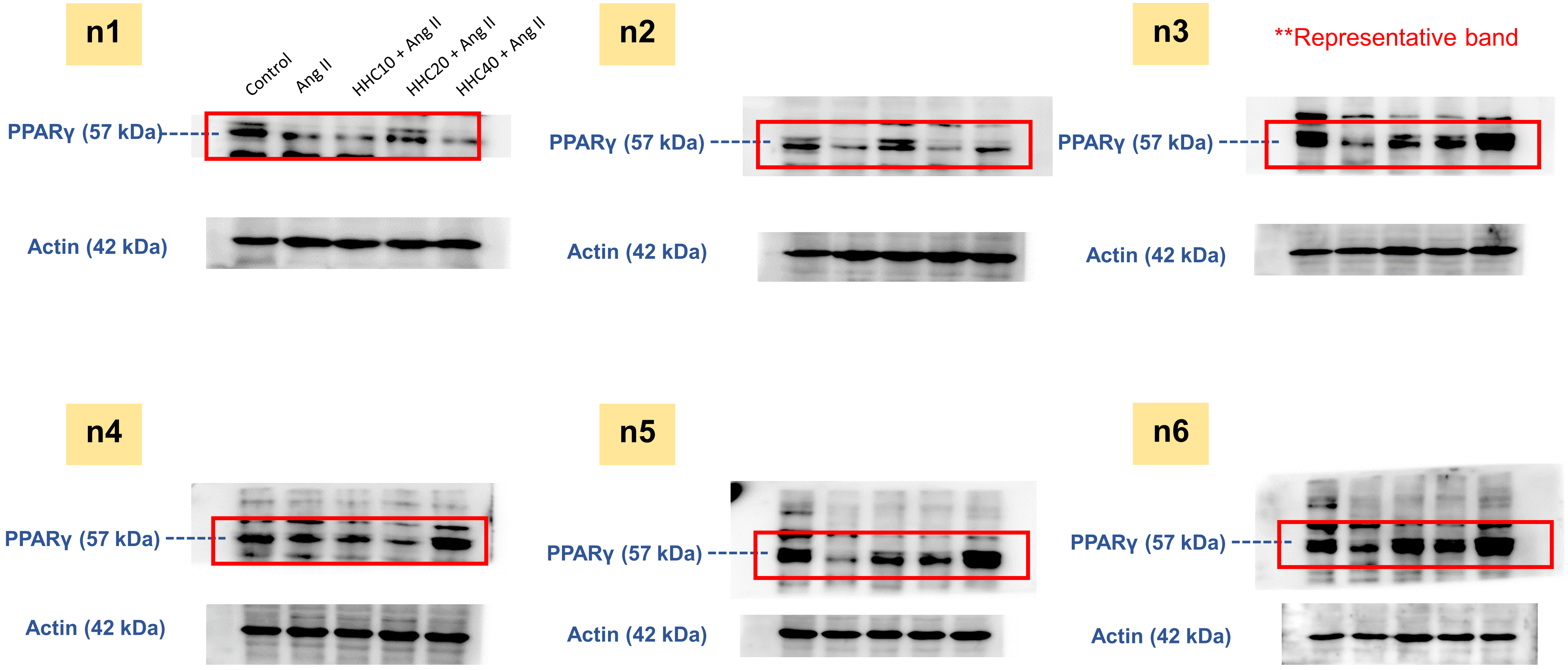

PGC-1α (90 kDa)

In figure 6

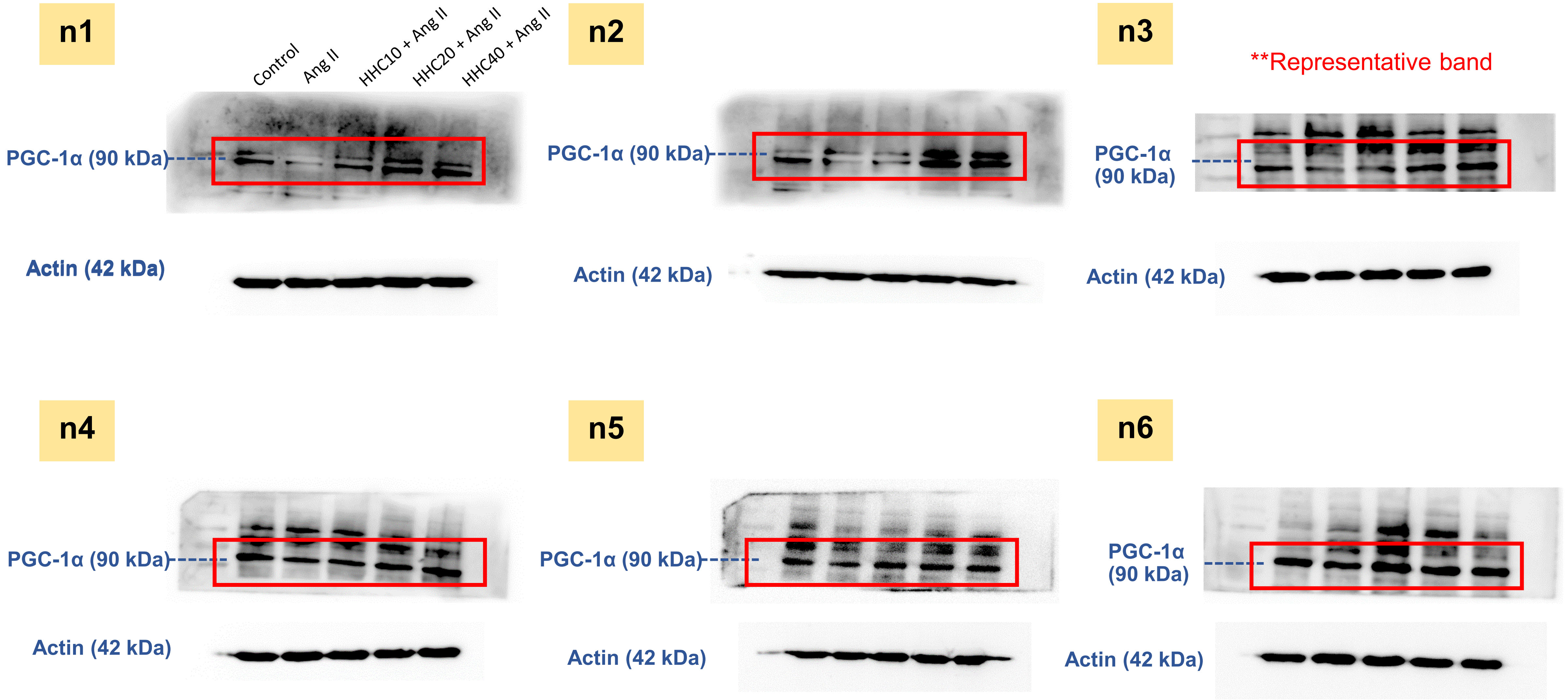

TNF-α (25 kDa)

In figure 7

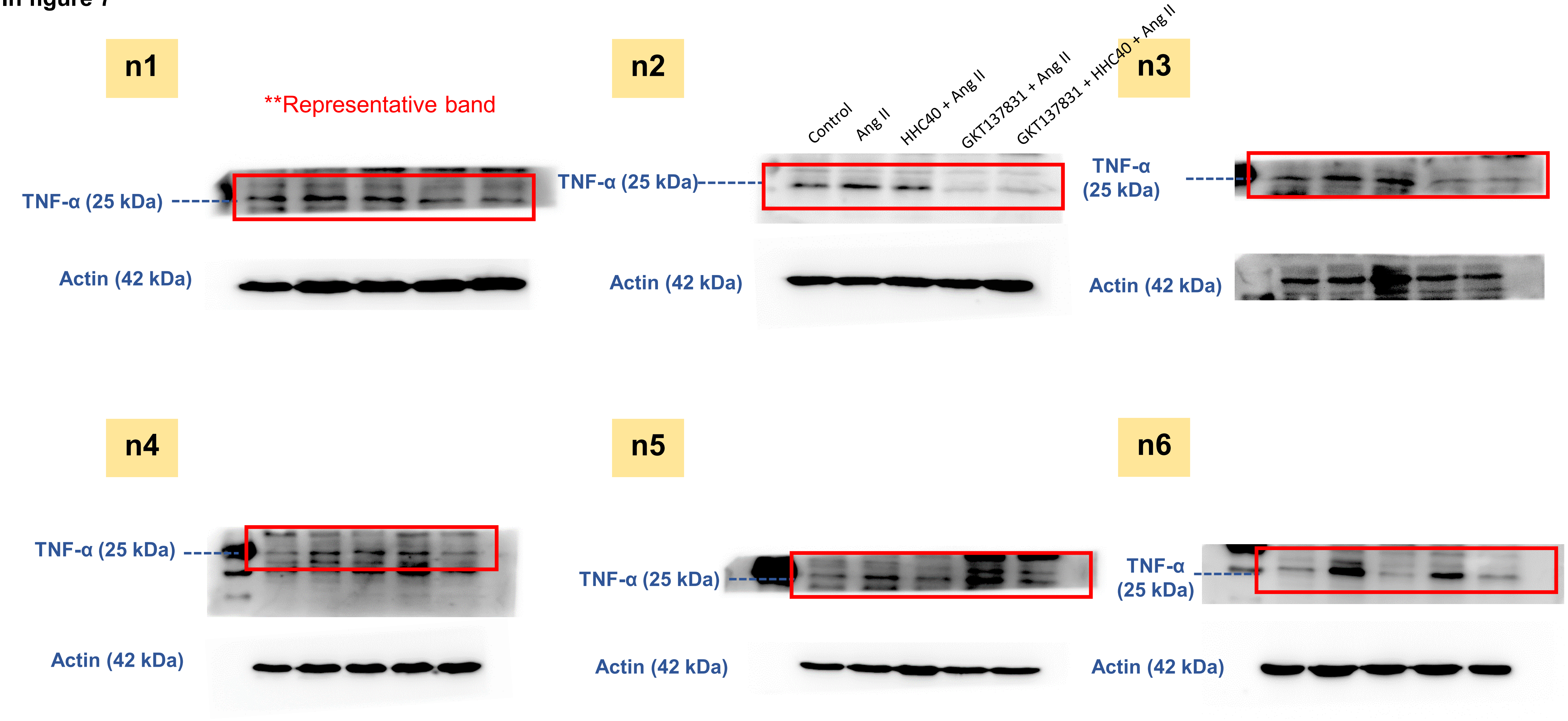

IL-6 (23 kDa)

In figure 7

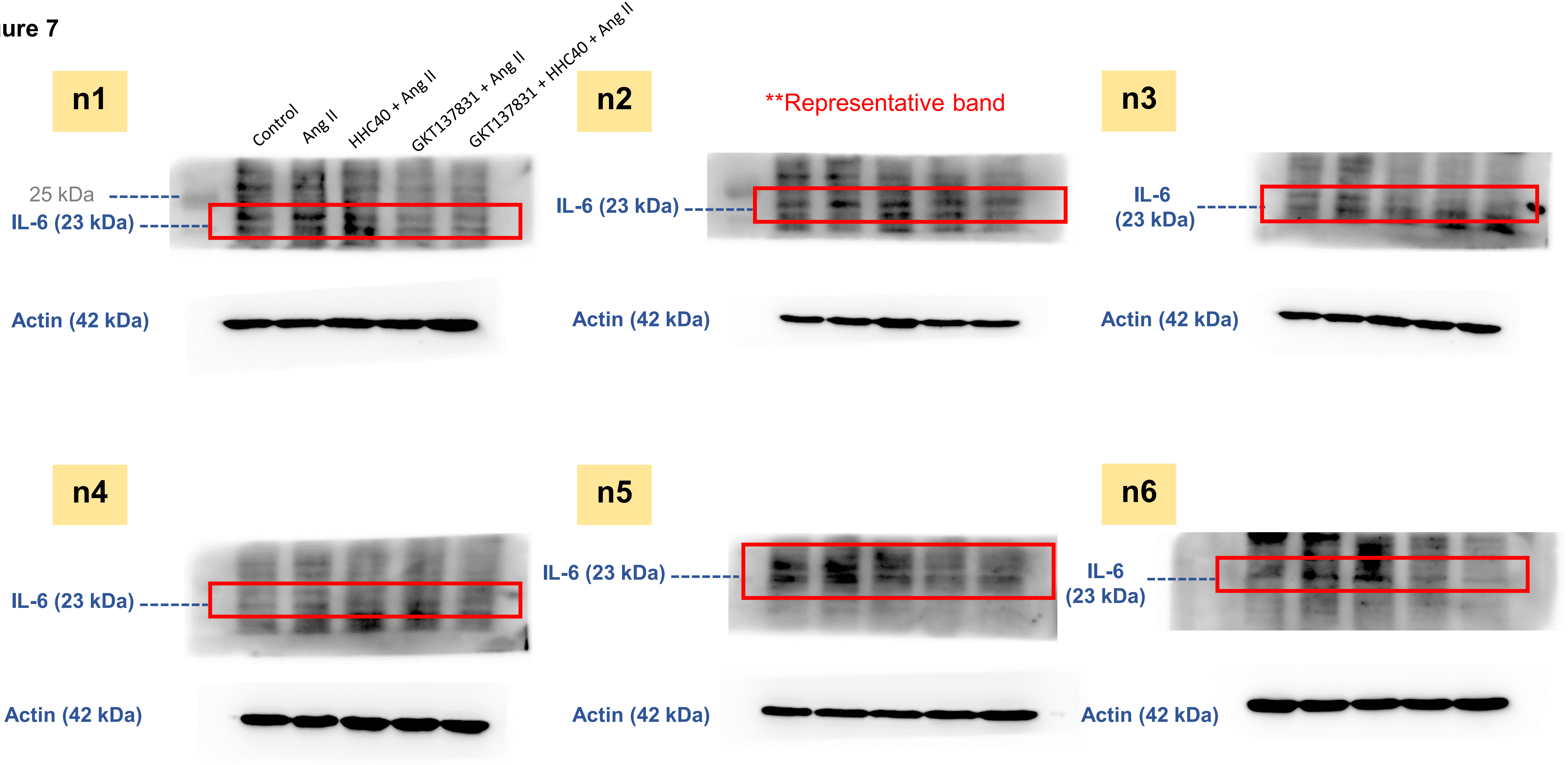

MMP9 (91 kDa)

In figure 7

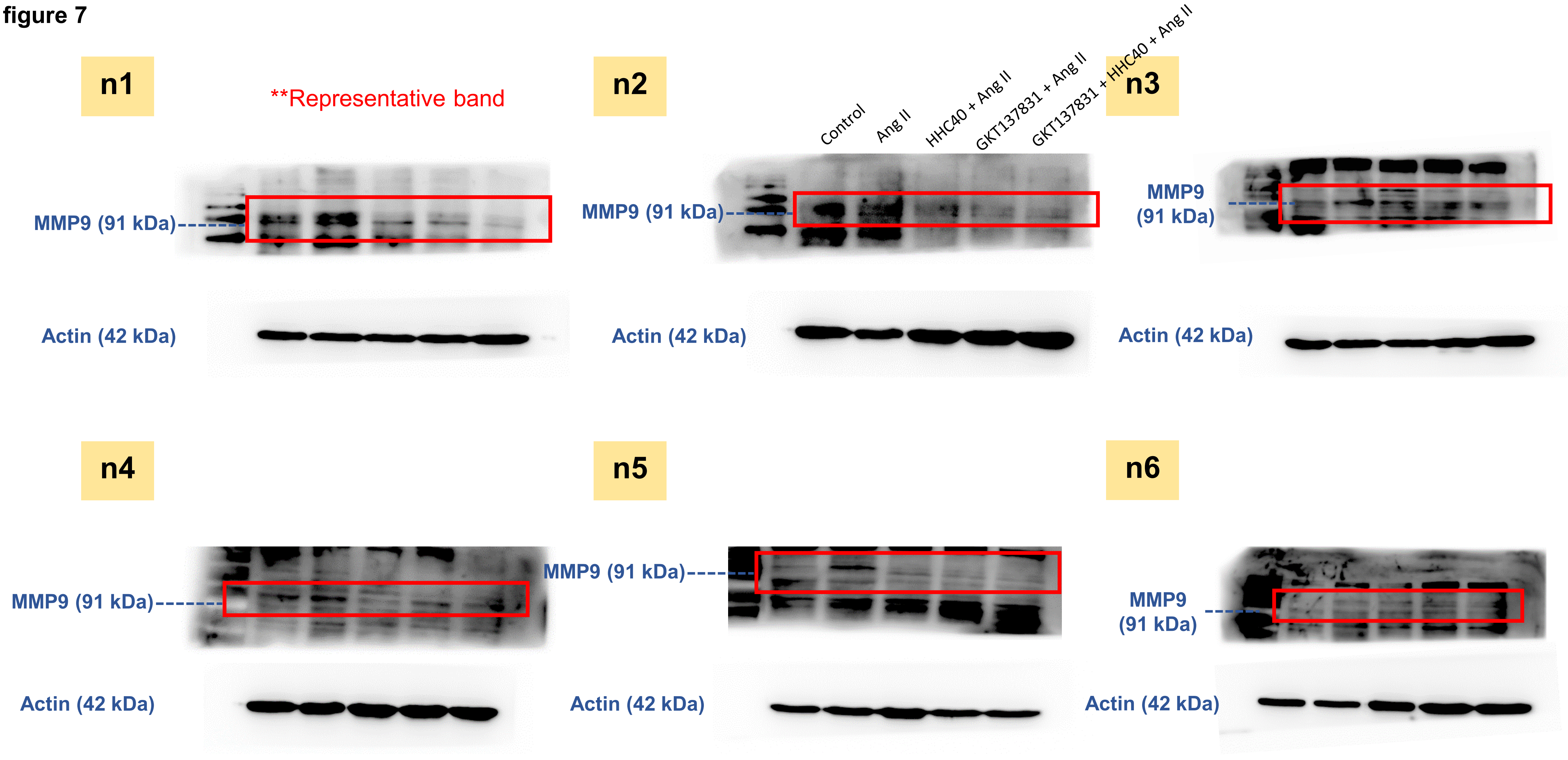

Supplement: Supplementary data western blots [file EXCLI-22-466-s-002.pdf]
